# Effects of Antithrombotic Drugs Fondaparinux and Tinzaparin on In Vitro Proliferation and Osteogenic and Chondrogenic Differentiation of Bone-Derived Mesenchymal Stem Cells

**DOI:** 10.1002/jor.21405

**Published:** 2011-03-22

**Authors:** Argiris Papathanasopoulos, Dimitrios Kouroupis, Karen Henshaw, Dennis McGonagle, Elena A Jones, Peter V Giannoudis

**Affiliations:** LIMM, Section of Musculoskeletal Disease, University of LeedsLeeds, United Kingdom

**Keywords:** low molecular heparins, bone healing, MSC, chemical thromboprophylaxis

## Abstract

An unexpected side effect of some classes of anticoagulants has been osteoporosis which may be, at least in part, related to deranged mesenchymal stem cell (MSC) function. The aim of the present study was to compare the effect of fondaparinux (FDP), a novel antithrombotic with a traditional widely used low molecular weight heparin, tinzaparin (TZP) on MSC proliferation and differentiation. MSCs were isolated from trabecular bone of 14 trauma patients by a collagenase-based digestion procedure and expanded in standard conditions until passage 3. Proliferation and differentiation of MSCs to chondrocytes and osteoblasts was assessed with or without the addition of FDP and TZP using standard in vitro assays and a broad range of drug concentrations. Flow cytometry was used for MSC phenotyping. In the age studied group (17–74 years old) the MSC frequency in collagenase-released fractions was 641/10^6^ cells (range 110–2,158) and their growth characteristics were ∼4 days/population doubling. Cultures had a standard MSC phenotype (CD73+, CD105+, CD146+, CD106+, and CD166+). Cell proliferation was assessed by both colony-forming unit-fibroblast (CFU-F) and colorimetric tetrazolium salt XTT assays. In both assays, MSC proliferation was inhibited by the addition of TZP, particularly at high concentrations. In contrast, FDP had no effect on MSC proliferation. Osteogenic differentiation and chondrogenic differentiation were not affected by the addition of either TZP or FDP. Whilst MSC proliferation, but not differentiation, is negatively affected by TZP, there was no evidence for adverse effects of FDP in this in vitro model system which argues well for its use in the orthopedic setting. © 2011 Orthopaedic Research Society Published by Wiley Periodicals, Inc. J Orthop Res 29: 1327–1335, 2011

A complication of fractures, deep vein thrombosis (DVT), leads to pulmonary embolism (PE) with up to a 28% DVT incidence being reported post-fractures.^1^ Tromboprophylaxis in patients undergoing surgery with unfractioned heparins (UFH) and, more recently, low-molecular-weight heparins (LMWHs) significantly reduce the risk of DVT.^2^–^4^ The use of anticoagulants and UFH in particular has been associated with systemic osteoporosis which is a known risk factor for poor fracture healing. Osteoporosis is endemic in developed and regardless the cause, osteoporosis results from superseding activities of osteoclasts compared to osteoblasts.^5^ Other factors associated with impaired healing of 5–10% of the million or so fractures seen in the UK each year^6^,^7^ are the absence of adequate mechanical stability, infection, diabetes, smoking, high alcohol intake, and pharmacological agents.^8^–^10^ With regard to the effect of LMWHs on bone metabolism, several animal and in vitro studies, have reported a negative effect on bone formation.^11^–^13^ It appears that the risk may be decreased by using LMWH instead of UFH^11^ but the effect is not abolished.^12^,^13^

Another anticoagulant that has been recently introduced in the clinical setting is fondaparinux (FDP), a novel antithrombotic that has the specific ability to inhibit factor Xa. In vitro studies^14^,^15^ have been performed comparing its effect on bone metabolism with previously used LMWHs (Enoxaparin and Dalteparin) and these have shown that FDP did not have a negative effect on human osteoblast proliferation in contrast to Enoxaparin and Dalteparin. However, to the best of our knowledge, no study has been performed to evaluate the effects on bone differentiation of another wide-used LMWH, Tinzaparin (TZP).

Mesenchymal stem cells (MSCs) are highly proliferative stromal cells that are capable of forming bone and cartilage both in vitro and in vivo and may play an important role in bone health by contributing to the osteoblast pool of cells. The aim of this study therefore was to investigate and compare the effects of FDP with TZP on proliferation and differentiation of MSCs derived from trabecular human bone.

## MATERIALS AND METHODS

### Preparations of Drugs

The antithrombotic drugs used in this study were FDP (GlaxoSmithKline 2.5 mg, Middlesex, UK), a new antithrombotic drug that inhibits factor Xa specifically, and TZP (LEO Pharmaceutical Products, 3,500 International Units, Buckinghamshire, UK), one of the most commonly used drugs for the prevention and treatment of DVT. The MSC response to FDP was studied at its peak plasma concentration obtained on doses most commonly used for prophylaxis after a fracture^14^ as well as at several higher concentrations (latter were used for conducting the tests because of the accumulation and resulting higher concentration in the bone in long-term therapy). TZP PC dosage was obtained from on-line Pharmacogenomics knowledge base (http://www.pharmgkb.org/do/serve?objId=PA451694&objCls=Drug). Stock solutions were prepared by diluting drugs in non-hematopoietic expansion medium (Miltenyi Biotec, Bisley, UK) to make the following dilutions: TZP 0.5 IU/ml (plasma concentration, PC), 5 IU/ml (×10) and 50 IU/ml (×100); FDP 0.34 µg/ml (PC), 3.4 µg/ml (×10) and 34 µg/ml (×100). Half-media changes with both drug preparations were performed every 48 h. Cells incubated in medium without drugs were used as controls.

### Cell Extraction from Trabecular Bone Pieces

MSCs were isolated from iliac crest bone chips obtained during trauma surgery. In total, 14 individuals participated in the study (Table 1). All patients were suffering from pelvic fractures but were otherwise healthy individuals. All patients provided informed consent while approval from the local ethics committee was obtained for all samples.

**Table 1:** Patient's Participation and Sample Characterization^a^

					Days			
					
Sample ID	Sex	Age	Cells per Sample, 10^6^	CFU-Fs per 10^6^ Cells	p0^b^	p0–p1	p1–p2	p2–p3
2	F	70	NT	NT	11	3	3	3
3	M	24	10	641	9	5	4	3
4	F	57	31	2,158	6	5	5	3
5	F	74	NT	NT	8	7	8	5
6	F	70	NT	NT	8	7	8	5
7	M	43	25	140	17	3	5	4
8	M	64	NT	NT	17	11	10	11
9	M	55	12	874	21	3	4	4
10	M	25	5	273	14	2	2	4
11	M	61	3	110	19	4	7	13
12	M	52	17	417	11	4	4	2
13	F	39	21	1,700	7	3	4	6
14	M	17	5	750	10	8	9	6
16	M	44	NT	NT	10	9	2	2
Median		54	12	641	11	5	5	4
Range		17–74	3–31	110–2,158	6–21	2–11	2–10	2–13

NT, not tested.

a Inclusion criteria: Age of 17 years or greater, adequate neurovascular status, absence of infection and systemic diseases (diabetes, rheumathoid arthritis, etc). Exclusion criteria: severe hepatic and renal impairment, pregnancy and lactation, non-unions resulting from bone diseases, dementia, pathological fractures, cancer, systemic treatment for osteoporosis.

b Initial confluent (∼80%) cultures were designated “passage 0” (p0) and subsequent passaging was performed as 1:2 splits until passage 3 (p3).

MSCs were extracted from bone chips by collagenase digestion technique as previously described.^16^,^17^ Briefly, ∼0.1 g bone pieces were transferred in Eppendorf microtubes containing 0.5 ml of 0.25% collagenase (Stem Cell Technologies, Vancouver, Canada) and cell extraction by tissue digestion was performed for 4 h at 37°C. Released cells were filtered through 70 µm Cell Strainer (BD Biosciences, San Jose, CA) and counted using Trypan Blue staining (Sigma, Dorset, UK). The average number of cells recovered per sample was 12 × 10^6^ (range 3–31 × 10^6^, Table 1).

### Standard Colony-Forming Unit-Fibroblast (CFU-F) Assay to Measure the Number of MSCs in Collagenase-Released Cell Fractions

One million collagenase-released cells was placed overnight into triplicate 25 cm^2^ tissue culture flasks in DMEM/10% FCS (Invitrogen, Paisley, UK) to allow adherent cell attachment, as described previously.^17^ Next day medium was changed to the non-hematopoietic expansion medium (Miltenyi Biotec). Cells were next grown in the NH expansion medium with twice-weekly half-media changes and stained with 1% w/v Crystal Violet (Sigma) on the 14th day of culture as previously described.^18^ Half-media changes were performed so culture conditions would not be dramatically altered after each feeding, by retaining stable levels of soluble endogenous-produced growth factors and proteins. Individual colonies were scored by microscopic examination at low power (×50). Antibiotics 1% penicillin/streptomycin (Invitrogen) were used in all in vitro experiments.

### Isolation and Expansion of Collagenase-Released MSCs

For this, 20 × 10^6^ collagenase-released cells were similarly adhered overnight in 25-cm^2^ flasks in DMEM/10% FCS. Next day, adherent cells were placed in 5 ml of non-hematopoietic expansion medium and cultures were grown to confluence with twice-weekly half-media changes. Initial confluent (∼80%) cultures were designated passage 0 (p0) and subsequent was performed as 1:2 splits until passage 3 (p3). For passaging MSC cultures were lifted off using Trypsin-EDTA solutions (Invitrogen) as previously described.^17^,^19^ Proliferation and differentiation assays were performed using p3 cultures.

### Flow Cytometry to Confirm the MSC Nature of Bone-Derived Cultures

MSC marker phenotyping was performed on p3 cultures as previously described.^18^,^19^ CD13-FITC and CD105-PE were from Serotec (Kidlington, UK), CD45-FITC was from DAKO (Ely, UK), CD106-PE, CD73-PE, CD146-PE, and CD166-PE were from BD Bioscience (Oxford, UK), and all isotype-specific negative controls were from Serotec. Three-color flow cytometry was performed on a BD FACScan. Dead cells were gated out based on propidium iodide exclusion (Sigma).

### Analysis of MSCs Proliferation

The effect of drugs on MSC proliferation was performed using CFU-F and XTT assays on p3 cultured MSCs as optimized in our previous studies.^20^,^21^ In CFU-F assay, 500 p3 MSCs were initially seeded into triplicate wells of six-well plates in DMEM/10% FCS to allow overnight adherence without drug interference. Next day media was changed to an appropriate drug-containing or control expansion media and cells were grown for further 7 days. In the treatment group a broad of drug concentrations, from PC to 100-fold higher was used to determine age-dependent effects and in control group MSCs were expanded normally. MSC proliferation was assessed by manual counting of colonies formed 7 days after plating as previously optimized.^17^,^21^ Colonies were stained with Crystal violet and triplicates were averaged for each treatment group. Additionally, the number of cells per colony was counted for 10 random colonies in each treatment group and averaged. In the XTT assay cell proliferation was indirectly assessed by measuring metabolically active cells, as optimized previously.^20^,^21^ In this assay 500 p3 MSCs were seeded in quadruplicate wells of 96-well plates in DMEM/10%FCS. Similarly to CFU-F assay, media was changed 24 h later to drug-containing expansion media and MSCs were allowed to grow for further 7 days. Optical density was measured after the addition of the XTT reagent (Roche Diagnostics, Welwyn Garden City, UK). Inhibition of MSC proliferation was assessed as a reduction in optical density (OD) compared to no-drug control.^20^,^21^

### Osteogenic Differentiation Assay

Alkaline phosphatase (ALP) assay, Alizarin Red (AR) staining for mineralized matrix and calcium production assay were used to evaluate the osteogenic potential of MSCs. In all experiments osteogenesis was induced in 3 × 10^4^ p3 MSCs using commercial osteoinductive medium OsteoDiff (Miltenyi Biotec). The effects of FDP and TZP were compared using cells from the same four donors (aged 17–52). MSCs were initially adhered in triplicate 35-mm round dishes in DMEM/10% FCS and next day media was changed to OsteoDiff with or without appropriate dilutions of drugs. Differentiation was induced for a maximum of 3 weeks with half-media changes twice a week. ALP, AR, and calcium assays were performed as previously described.^18^,^21^ Briefly, ALP staining was performed on day 14 post-induction using Fast RR Blue Salt (Sigma). Calcium was extracted on day 21 post-induction using 1 ml of 0.5 N HCl/well and measured using Ca^++^ assay (Thermo, Cramlington, UK) and Calcium/Phosphorus standard (Sigma). The purple color was read using 570 nm filter on a GENios Microplate Reader. For AR assay, cells were fixed with cold ethanol and stained with AR (pH 4.1) at room temperature (RT). After five washes in distilled water, dishes were air-dried and scanned. All assays were performed in triplicate for each drug dilution.

### Chondrogenic Differentiation Assay

In this assay, 0.25 × 10^6^ p3 MSCs were initially pelleted down in quadruplicate 1.5 ml Eppendorf tubes and placed in 0.5 ml of commercial chondroinductive ChondroDiff medium (Miltenyi Biotec) with or without drugs. Caps were loosened to allow air exchange. Cell pellets were fed three times per week with half-medium changes. On day 21, three pellets were assayed for sGAG content after digestion with 1 mg/ml Papain (Sigma,) using a commercially available assay (Wieslab AB, Boldon, UK) as previously described.^18^,^21^ The blue color was read using 630 nm filter on a GENios Microplate Reader. A standard curve was built using assays' calibrators of known sGAG content (included in the kit). The remaining pellet was processed for staining with 1% Toluidine blue (Sigma), as previously described.^18^,^19^

### Statistical Analysis

All calculations were done on using the SPSS software standard version 13.0 for Windows. Data are expressed as means (standard deviation) or median (range) as appropriate. Matched paired data (without and with the addition of drugs) were compared using nonparametric Wilcoxon paired test and Spearman test was used for testing age-dependency. The cut-off value for significance was *p* = 0.05.

## RESULTS

### Characterization of Bone-Derived MSCs

As seen in Table 1, the concentration of MSCs (measured by the CFU-F assay) in the collagenase-released cellular fraction was not age-dependent and within previously published ranges.^17^ The proliferation kinetics of MSCs was measured up to p3 and the average time required for MSCs to reach p0 was 10 days. The population doubling time between subsequent passages was on average 4 days (range 3–6 days), consistent with our previously published data for similar age group.^17^

**Figure 1:**
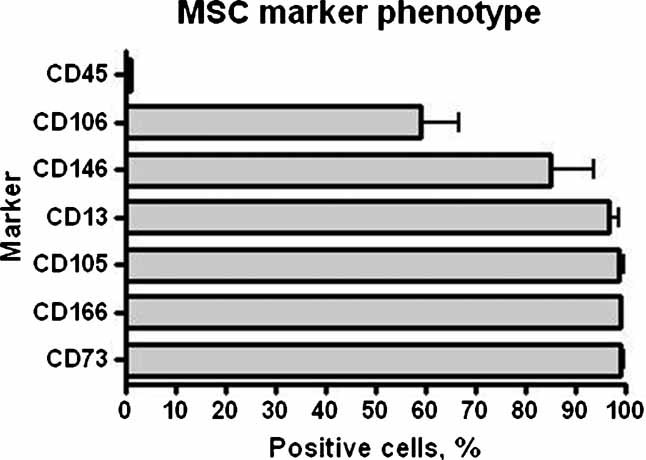
The marker phenotype of MSC cultures grown from human trabecular bone. Error bars represent SD (*n* = 5 donors, selected to cover age range 17–70 years old). The results show cell surface phenotype consistent with MSCs (CD73+, CD105+, CD166+, CD146+, CD106+, CD13+, and CD45−).

The procured cultures possessed adipogenic differentiation capacity (data not shown) and had a surface phenotype consistent with MSCs: a mean of 99% cells positive for CD73, 98.5% cells positive for CD105, 99% positive for CD166, 85% positive CD146, 59% positive for CD106, 96.5% positive for CD13 and negative for CD45 (Fig. 1). Therefore, p3 bone-derived cultures had growth kinetics and the phenotype consistent with MSCs.

### The Effects of TZP and FDP on MSC Proliferation

**Figure 2:**
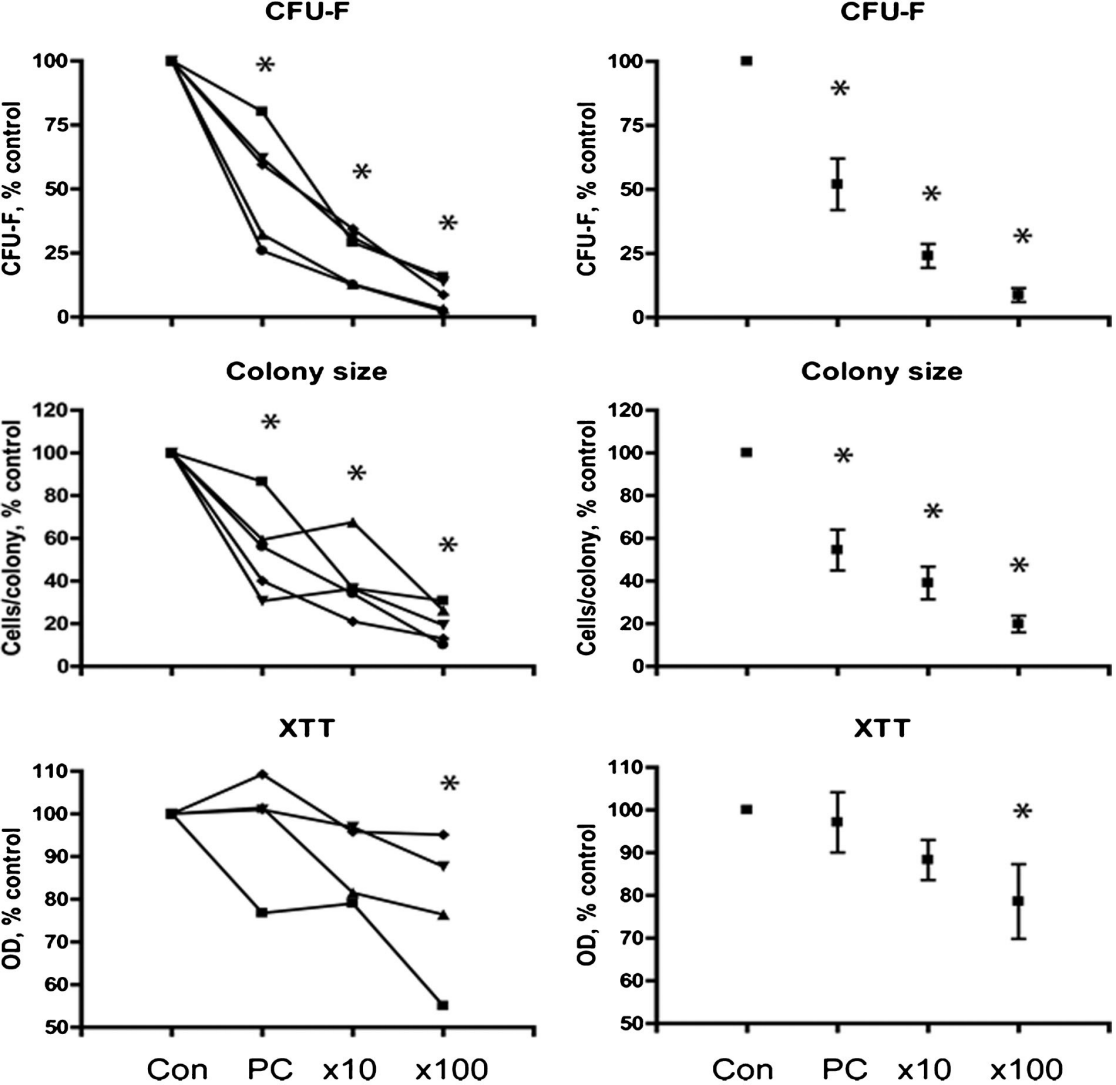
The effects of TZP on MSC proliferation using the CFU-F assay (top and middle panels) and XTT assay (bottom panels). Individual donor-derived cultures are shown on the left and combined results for all donors (mean ± SD) are shown on the right. Dose-dependent inhibition of MSCs proliferation is observed using both assays. *p < 0.05.

**Figure 3:**
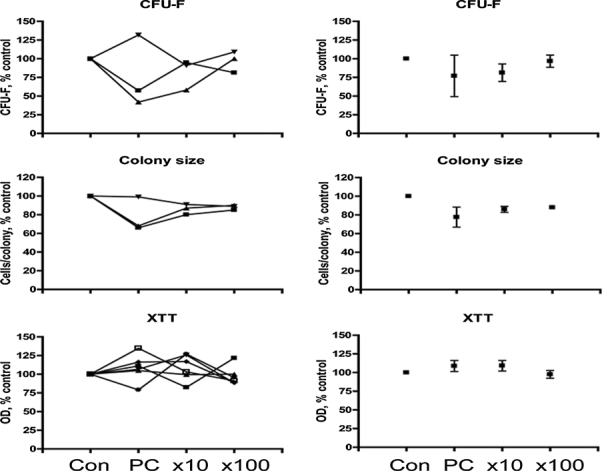
The effects of FDP on MSC proliferation using the CFU-F assay (top and middle panels) and XTT assay (bottom panels). Individual donor-derived cultures are shown on the left and combined results for all donors (mean ± SD) are shown on the right. No dose-dependent inhibition of MSCs proliferation is observed using both assays.

The effects of drugs on MSC proliferation were assessed using a standard CFU-F assay on cultured MSCs and the XTT assay.^20^,^21^ Although CFU-F assay remains fairly subjective, it accurately measures the effects of the compounds on the most proliferative cells.^21^ The average CFU-F of p3 MSCs was 6.5% (range 2.2–12.2%, *n* = 6), consistent with previously published data^17^ indicating that the remainder were less-proliferative progenitor cells.^22^ On the other hand, XTT assay provides highly reproducible read-outs, but the results represent the effects of compounds on both highly proliferative and less-proliferative cells, as XTT binds to all metabolically active cells. In combination, these assays accurately reflect the effects of the compounds on MSC proliferation in the whole culture. The results for both assays are presented as a percent inhibition of CFU-F formation or inhibition of ODs, compared to “no drug” controls (Figs. 2 and 3). Although the dose of drugs used at the cellular level and the growth kinetics of the cells in the culture wells could be different between CFU-F and XTT assay, similar trends were observed.

As shown in Figure 2, MSC proliferation was inhibited by TZP. CFU-F assay revealed statistically significant inhibition (*p* < 0.05) at all concentrations of TZP, whereas in the XTT assay statistically significant inhibition (*p* = 0.036) was achieved at the highest concentration of TZP (X100). The size of colonies in the CFU-F assay, measured as the number of cells per colony, was also inhibited by media supplementation with TZP (Fig. 2). In contrast, MSC proliferation was not affected by the addition of FDP, at any concentration of the drug, as evidenced by both CFU-F and XTT assays (Fig. 3). Altogether these findings showed that there was some considerable inhibition of MSC proliferation by the addition of TZP, particularly at high concentrations. In contrast, FDP had no effect on MSC proliferation.

### Osteogenic Differentiation

**Figure 4:**
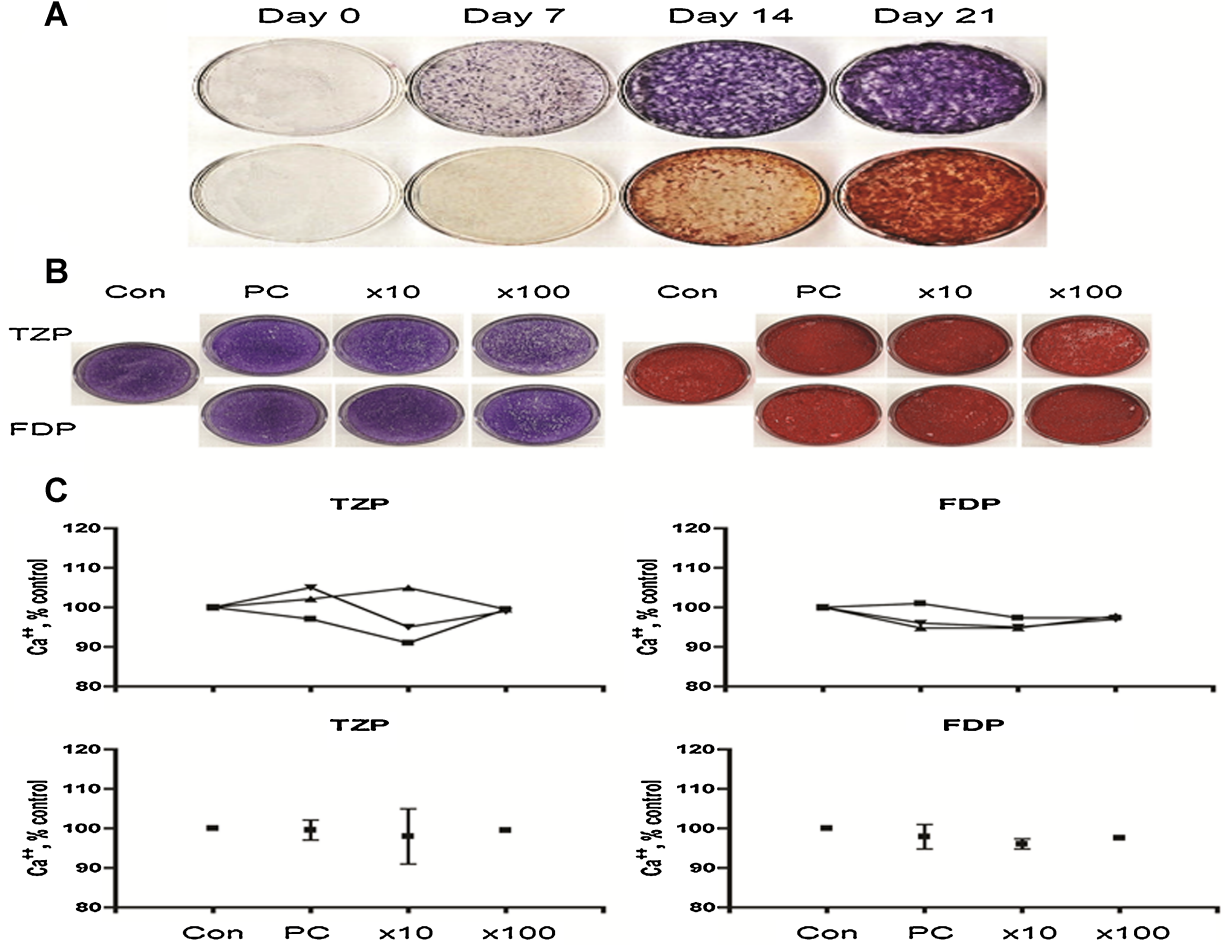
The effects of TZP and FDP on MSC osteogenesis. (A) Osteogenesis assay time course in control p3 MSCs. Top panel, ALP activity; bottom panel, Alizarin Red staining. (B) Day 14 ALP activity (left) and day 21 Alizarin Red staining (right) with the addition of FDP and TZP. (C) Day 21 calcium production. Top panel shows individual donor-derived cultures and bottom panel shows combined results for three donors (mean ± SD). No effect of both drugs on ALP activity and mineralization was found. Representative experiments are shown for ALP and Alizarin Red staining. [Color figure can be seen in the online version of this article, available at http://wileyonlinelibrary.com/journal/jor]

The effects of FDP and TZP on MSC osteogenic differentiation were assessed using p3 MSCs. A negative trend was observed between the age of donor and calcium production by p3 MSC cultures (*r*^2^ = −0.6, *p* = 0.07) and to avoid age-dependent effects, FDP and TZP were tested using cells from the same four donors. ALP activity and Calcium production were assessed at their peak levels on days 14 and 21, respectively (Fig. 4A). As seen in Figure 4B, the addition of both FDP and TZP in osteogenic medium did not affect ALP activity. Similarly, the addition of TZP or FDP to OsteoDiff showed no inhibition of mineralization, evidenced by AR staining (Fig. 4B). Consistent with ALP and AR staining, calcium production was not affected by the addition of neither TZP nor FDP (Fig. 4C). Altogether, these results demonstrated no deleterious effects of TZP or FDP concentrations on the osteogenic differentiation of MSCs in vitro.

### Chondrogenic Differentiation

**Figure 5:**
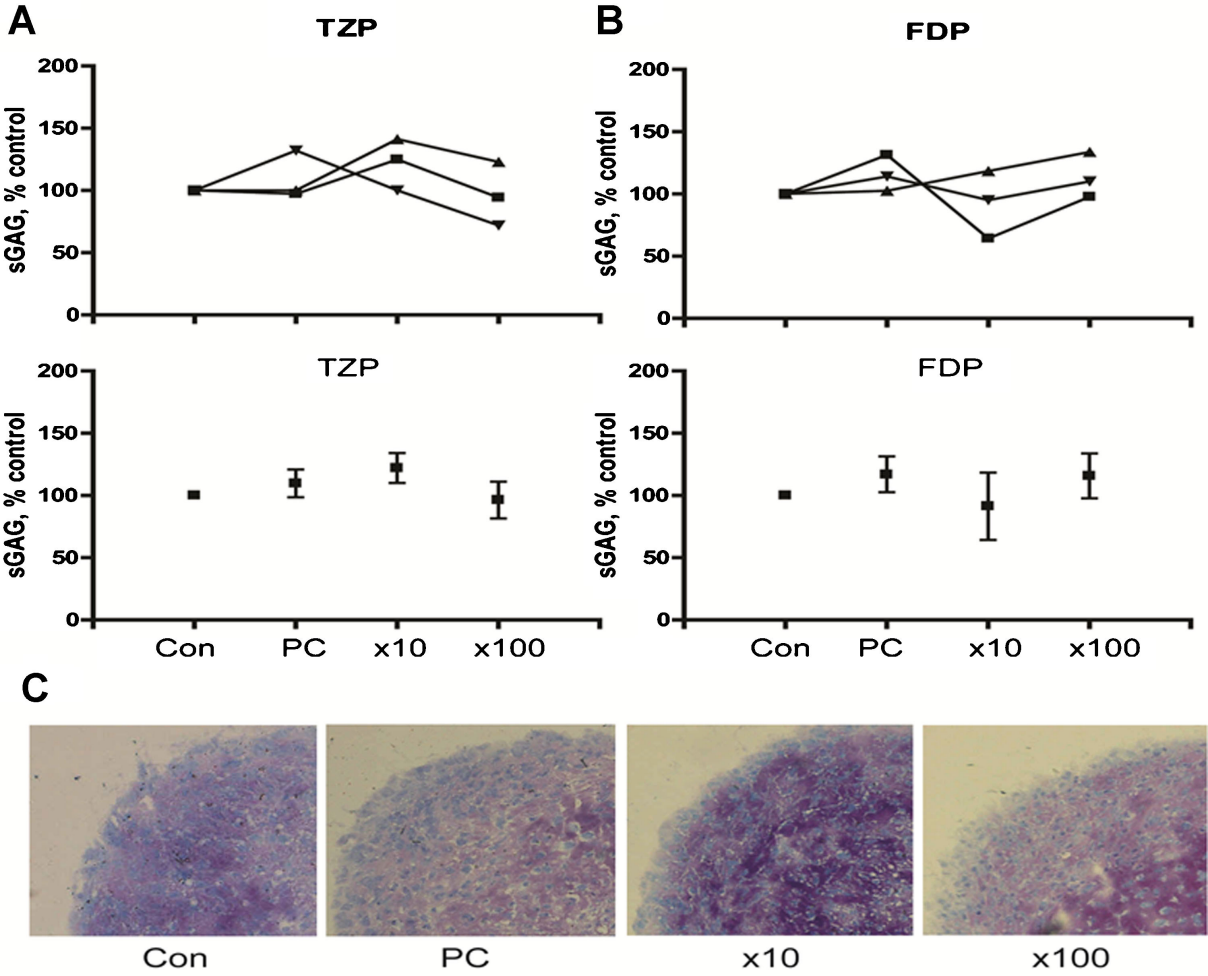
The effects of TZP and FDP on MSC chondrogenesis. Day 21 sGAG production by p3 MSCs grown as pellets in ChondroDiff supplemented with different concentrations of TZP (A) and FDP (B,C). Individual donor-derived cultures are shown at the top and combined results for all donors (mean ± SD) are shown at the bottom. No effect on sGAG production was found using both drugs. Toluidine Blue staining of chondrogenic pellets in a representative experiment with the addition of FDP is shown in (C). [Color figure can be seen in the online version of this article, available at http://wileyonlinelibrary.com/journal/jor]

The average sGAG production p3 MSC cultures on day 21 post-induction was 4.5 µg/pellet (range 3.8–16.6, *n* = 3 donors), consistent with previously published results.^20^ As shown in Figure 5A, no statistically significant decrease in the sGAG/pellet was observed if media was supplemented with TZP. Similar results were obtained for ChondroDiff supplemented with FDP (Fig. 5B). Figure 5C illustrates the lack of chondrogenesis inhibition by FDP by staining chondrogenic pellets with Toluidine Blue. Altogether, these data showed that neither TZP nor FDP had any effect on chondrogenic differentiation of MSCs in vitro supporting the idea that these molecules are unlikely to affect the endochondral ossification pathway which is important in fracture repair.

## DISCUSSION

In the present work we compared the effects of FDP and a widely used LMWH, TZP on MSC function in vitro. To the best of our knowledge, parallel comparison of these two drugs using the same donor-derived cells has not been previously performed. Our results revealed that neither anticoagulants affected MSC osteogenesis and chondrogenesis. However unlike TZP, FDP did not inhibit MSC proliferation and hence was superior to TZP in terms of its effects on MSC proliferation potential in vitro.

Previous in vitro experiments have documented negative effects of some LMWHs on human osteoblasts. One study showed the superiority of FDP compared with other LMWHs Dalteparin and Enoxaparin in respect to mitochondrial activity and protein synthesis by osteoblasts in vitro.^15^ Enoxaparin and Dalteparin, on the other hand, led to a significant decrease of matrix collagen content and calcification by osteoblasts, in concentrations equal or higher than the therapeutic dose. Of note, osteoblast cultures in the above study were derived from a distal femur bone of elderly patients with arthritis. In our study, the effects of FDP and TZP were investigated on the osteoblast precursors, namely MSCs, generally in a younger population group. The choice of MSCs as target cells was relevant to a clinical situation of fracture when both proliferation and correct differentiation of immature MSCs is needed for a biological healing cascade to occur in a timely and efficient manner.^23^ In the first part of our study we indeed confirmed that cultures derived from normal trabecular bone had growth characteristics and the phenotype consistent with MSCs.^16^,^17^,^24^,^25^ We acknowledge that further work is needed to establish the effects of FDP and TZP on osteoblasts, and not only MSCs.

Handschin et al.^14^ reported a significant dose-dependent inhibition of osteoblast proliferation following the administration of Dalteparin to human osteoblasts in vitro whereas no inhibitory effects were observed in the FDP treated group. It is noteworthy that unlike other studies focused on total number of osteoblasts in culture, Handschin et al. investigated osteogenesis by reverse transcriptase-polymerase chain reaction for the expression of osteogenesis-related genes (osteocalcin, collagen type I, and ALP). In our study we used long-term functional assays of osteogenesis and chondrogenesis and were able to measure the effects of FDP and TZP on MSC differentiation in a quantitative manner. FDP was found to have no deleterious effects on MSC osteogenesis. Furthermore, our study provided first evidence that FDP was well tolerated not only by osteoblasts, but also by MSCs. Previously we showed inhibitory effects of NSAIDs on the MSC chondrogenesis using similar functional assays and it appears that these assays represent a robust way of assessing pharmacological agents relevant to orthopedics in vitro.^20^ In this study we performed end point differentiation assays and did not test the effects of drugs throughout the culture period, which could have been more informative, but necessitated significant expansion of cells leading to the loss of potency.^26^ Small donor group size for osteogenesis and chondrogenesis represents another limitation of this study. On the other hand, the fact that we used several independent assays for proliferation, osteogenesis, and chondrogenesis assured that the observed effects were real and not dependent on the choice of assay used.

Previous and our findings therefore support the view that there is no adverse effect of FDP on human osteoblasts and MSCs in vitro. Both FDP and TZP are prepared by different methods of depolymerization and differ to some extent in their pharmacokinetic properties and anticoagulant profiles. FDP is a synthetically produced pentasaccharide sequence with a mean molecular weight of 1.728 and half life 17 h, with only anti-Xa activity and no anti-IIα activity.^27^ TZP has a higher molecular weight (4.800), half life ∼4 h, and 1.6 anti-Xa/anti-IIa ratio.^28^ The effect of heparin and its derivatives on osteoblast differentiation are molecular weight dependent.^29^ Molecular size has been reported to affect heparin's affinity for heparin-binding plasma proteins, as well as endothelial cells and platelets.^30^ Although less pronounced than UFH, TZP shows significant binding to endothelial cells and osteoblasts.^31^ Osteoblast proliferation is influenced by insulin like growth factors (IGFs)^32^ and human osteoblasts express a surface binding for IGFs to which TZP is able to bind and to compete with IGF. This might explain the impaired regulation of proliferation and differentiation of osteoblasts^33^ and, potentially, MSCs. In contrast, FDP lacks the modulatory effects of heparin.^34^

Moreover, many growth factors and cytokines thought to modulate vascularization during fracture healing (fibroblast growth factor, vascular endothelial growth factor, platelet derived growth factor) are heparin dependent.^35^ Thus, administration of TZP may alter growth factor bioavailability and activity. It seems possible that interaction of TZP and FDP with osteoblast is either quantitatively or qualitatively different and this reflects the differences in molecular weight distribution of the two preparations.

In our study a broad range of drug concentrations, from PC to 100-fold higher than their peak PC were used to determine dose-depended effects. MSC proliferation and differentiation were assessed using several parallel end-point studies. In the CFU-F assay, the direct measurements of the effects of drugs on most proliferating cells were studied. In the XTT assay, the reduction of a tetrazolium component (XTT) into soluble formazan product by the mitochondria of all viable cells was measured. Smaller inhibition effects upon the addition of TZP in the XTT assay compared to CFU-F assay could be explained by above factors or differences in cell density. The reduced MSC proliferation following TZP treatment could have implications for the initial expansion of the MSC pool during initial stages of fracture healing. This decrease could further delay the physiological events during endochondral and intramedullar ossification during healing processes. Using several independent assays for osteo- and chonrogenesis, no inhibition of MSC differentiation upon the addition of TZP or FDP was found. Altogether, these findings showed that TZP could be potentially detrimental during early but not late stages of fracture healing. FDP on the other hand would not be inhibiting MSC functionality during the whole fracture healing processes.

In conclusion, this work showed that FDP had no adverse effects on either MSC proliferation or osteogenic and chondrogenic differentiation in vitro. This is reassuring with respect to fracture repair and bone metabolism but needs ongoing clinical vigilance and observation to confirm that it is associated with skeletal health maintenance. Future studies may include clinical studies of prospective randomized nature, comparing FDP with other commonly used anticoagulants.
